# Collective Motion of Repulsive Brownian Particles in Single-File Diffusion with and without Overtaking

**DOI:** 10.3390/e20080565

**Published:** 2018-08-02

**Authors:** Takeshi Ooshida, Susumu Goto, Michio Otsuki

**Affiliations:** 1 Department of Mechanical and Physical Engineering, Tottori University, Tottori 680-8552, Japan; 2 Graduate School of Engineering Science, Osaka University, Toyonaka, Osaka 560-8531, Japan

**Keywords:** caged dynamics, stochastic processes, collective motion, single-file diffusion, normal and anomalous diffusion, displacement correlation, overtaking, hopping rate, label variable, Dean–Kawasaki equation

## Abstract

Subdiffusion is commonly observed in liquids with high density or in restricted geometries, as the particles are constantly pushed back by their neighbors. Since this “cage effect” emerges from many-body dynamics involving spatiotemporally correlated motions, the slow diffusion should be understood not simply as a one-body problem but as a part of collective dynamics, described in terms of space–time correlations. Such collective dynamics are illustrated here by calculations of the two-particle displacement correlation in a system of repulsive Brownian particles confined in a (quasi-)one-dimensional channel, whose subdiffusive behavior is known as the single-file diffusion (SFD). The analytical calculation is formulated in terms of the Lagrangian correlation of density fluctuations. In addition, numerical solutions to the Langevin equation with large but finite interaction potential are studied to clarify the effect of overtaking. In the limiting case of the ideal SFD without overtaking, correlated motion with a diffusively growing length scale is observed. By allowing the particles to overtake each other, the short-range correlation is destroyed, but the long-range weak correlation remains almost intact. These results describe nested space–time structure of cages, whereby smaller cages are enclosed in larger cages with longer lifetimes.

## 1. Introduction

Particles in dense liquids are hindered from free motion, being constantly pushed back by their neighbors. This is often described as a “cage” that confines each particle. The cage effect makes the motion subdiffusive and, in certain cases, leads to the glass transition [1,2].

**Figure 1:**
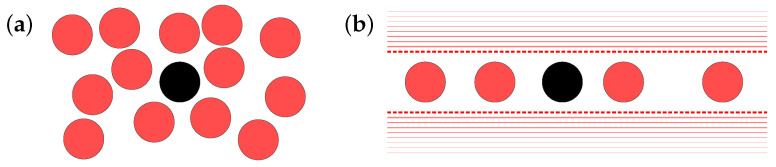
(**a**) Schematic description of a cage in a dense liquid, consisting of the surrounding particles that hinder free motion of the enclosed particle. (**b**) A (quasi-)one-dimensional model of the cage effect, with the particles confined in a narrow channel.

To be specific, let us consider a system consisting of Brownian particles with a nearly hardcore interaction. The position vector of the *i*-th particle, $r_{i}=r_{i}\left(t\right)$, is governed by the Langevin equation

(1a) $$m{\ddot{r}}_{i}=-\mu {\dot{r}}_{i}-\frac{\partial U}{\partial r_{i}}+\mu f_{i}\left(t\right),$$

with *m* and μ denoting the mass and the drag coefficient of the particle, $\mu f_{i}\left(t\right)$ representing the thermal fluctuating force, and the interaction being prescribed as

(1b) $$U=U\left(r_{1},r_{2},\ldots \right)=\sum_{\left(j,k\right)}V_{jk}$$

in terms of the pair potential $V_{jk}$. Among the fundamental statistical quantities characterizing this system is the mean square displacement (MSD), i.e., the second moment of the displacement $R_{i}=r_{i}\left(t\right)-r_{i}\left(0\right)$. If the interaction through *U* is negligible, each particle diffuses freely so that $\langle R_{i}^{2}\rangle$ grows in proportion to *t* (for timescales longer than m/μ). This occurs when the colloidal fluid modeled by Equation (1) is dilute enough. In contrast, particles in a denser colloidal fluid are hindered from free motion by the cage effect, so that the growth of $\langle R_{i}^{2}\rangle$ is much slower. As is illustrated in Figure 1a, every particle in such a system is almost arrested in a “cage” consisting of its neighbors. In extreme cases, the system ceases to be fluid and becomes a kind of amorphous solid, referred to as colloidal glass [1].

Although it is true that the cage effect suppresses the growth of MSD on the whole, the details are rather complicated [2]. The behavior of the MSD in dense liquids reflects at least three aspects of caged dynamics: nearly free motion within the cage for a short time, possible drift of the cage enclosing the particle at a longer timescale, and hopping of the particle out of the cage as a rare event. Proper characterization of these processes requires space–time description, typically in terms of some four-point space–time correlation [2,3,4], as the cage effect actually emerges from many-body dynamics involving collective motions of numerous particles correlated both spatially and temporally.

In search of insight into the theoretical treatment of such collective motions, here, we take note of the one-dimensional (1D) system illustrated in Figure 1b, following several authors who studied it as a simplified model of the cage effect [4,5,6,7,8,9,10]. The slow dynamics of such a 1D system are known by the name of single-file diffusion (SFD). In what we call the ideal SFD, every particle is eternally trapped within the “cage” formed by their neighbors. The MSD in the ideal SFD is known to grow subdiffusively as $\langle R_{i}^{2}\rangle \propto \sqrt{t}$ [11,12,13,14] (for 1D systems, we write $R_{i}$ instead of $R_{i}$). The subdiffusion in SFD emerges from collective motion of particles [5,10,15] and is also detected as a negative longtime tail in the velocity autocorrelation [16,17], indicating that the particle is pushed back by its neighbors. The importance of the collective motion is understood by considering the origin of the effective stochastic equation for a single particle in SFD: the one-body equation (yielding the negative velocity autocorrelation) is actually based on the collective dynamics described in terms of the fluctuating density field [18].

Focusing on the collective motions in SFD, the group of the present authors has noticed the usefulness of the *displacement correlation* $\langle R_{i}R_{j}\rangle$ [4,10,19]. It is a kind of four-point space–time correlation that probes both the time scale and length scale; the definition of the displacement includes *t*, while the spatial scale is included as the mean distance between the two particles (i,j). In the ideal SFD in which the particles are forbidden to overtake each other, the displacement correlation has been calculated both analytically and numerically [4,10,20]. The calculated displacement correlation revealed collective motions behind the slow diffusion in SFD, in contrast to free diffusion in which $\langle R_{i}R_{j}\rangle$ vanishes (unless i=j). The formalism for analytical calculation of the displacement correlation can be extended to the case of two-dimensional (2D) colloidal liquids [10,21], which reproduces some numerical findings in 2D systems, such as vortical cooperative motion, with negative velocity autocorrelation being a manifestation of the cage effect. One of the delicate points in this extension is that the cage effect in 2D liquids cannot be infinitely strong, in the sense that eventually the particles can escape from the 2D cage. The escape from the cage is an important process, which requires further investigation.

Methodologically, it should be noted that one of the most powerful approaches to SFD, employing the relation between the position of the tagged particle and the density fluctuation [10,12,17,22], has been formulated in reliance on the assumption that overtaking is completely forbidden. To extend this formalism to the case of “non-ideal SFD”—in which overtaking is allowed—is a challenging problem, which is the main objective of the present work. Most of the existing works on SFD with overtaking have reported numerical simulations [23,24,25,26,27], while analytical results are quite rare. In the exceptional case of lattice SFD, the method of vacancy dynamics [15,16] has been applied to quasi-1D geometries allowing some kind of overtaking [28,29]. The analysis of lattice SFD with overtaking, however, is not readily extensible to the cases with a continuous space coordinate.

In the present work, we discuss how the analytical results on the displacement correlation in SFD [4,10,20] are modified, if the particles are allowed to overtake each other and thereby escape from the quasi-1D cage as a rare event. The probability of the escape is regulated by the height of the potential barrier, denoted by $V_{\max}$, so that $V_{\max}\to +\infty$ and $V_{\max}\to 0$ correspond to the ideal SFD and the free diffusion, respectively. Some numerical solutions for finite $V_{\max}$ were included in our previous work [19], but analytical calculations were limited to the ideal case without overtaking, for the very reason that overtaking was difficult to take into account in the theoretical framework based on the density fluctuation. Now, the effect of a non-zero overtaking rate on the displacement correlation will be shown analytically as a main result of the present work.

Logical presentation of the main results in Section 4 requires a considerable amount of review in preparatory sections. For this reason, the paper is organized as follows: In Section 2, after the governing equation of the 1D system is specified and the collective motion is illustrated in a space–time diagram, we define some basic concepts and variables, such as the displacement correlation, overtaking, the fluctuating density field ρ(x,t), and the label variable ξ, clarifying their background. In particular, the kinematics of overtaking are discussed in SubSection 2.5. The usage of the label variable is a keystone for the analytical calculation of the displacement correlation [4,10,30], as is reviewed in Section 3 in the case of SFD without overtaking. The displacement correlation in this case is expressed in terms of a similarity variable, implying a nested space–time structure of cages. Subsequently, we proceed to the main topic in Section 4, in which we incorporate the effect of overtaking into the calculation of the displacement correlation by considering the dynamics of overtaking in terms of the $\Xi_{i}\left(t\right)$ prepared in SubSection 2.5. It is shown analytically and confirmed numerically that the infrequent overtaking events destroy the short-range correlation, while the long-range weak correlation remains almost intact. The final section is allotted for discussion and concluding remarks.

## 2. Formulation and Background

### 2.1. Specification of the System

We consider a 1D system of Brownian particles with short-range repulsive interaction, confined in a narrow channel, as is depicted in Figure 1b. With the position of the *i*-th particle denoted by $X_{i}=X_{i}\left(t\right)$, the system is governed by the 1D Langevin equation:

(2) $$m{\ddot{X}}_{i}=-\mu {\dot{X}}_{i}-\frac{\partial }{\partial X_{i}}\sum_{j<k}V\left(X_{k}-X_{j}\right)+\mu f_{i}\left(t\right),$$

where *m* and μ represent the mass and the drag coefficient of the particle, respectively. The system contains *N* particles within the length *L*. Posing the periodic boundary condition, $X_{i+N}=X_{i}+L$, we consider the limit of N→∞ with the density $\rho_{0}\overset{\mathrm{def}}{=}N/L$ kept constant.

The thermal fluctuating force, $\mu f_{i}\left(t\right)$, is characterized by the variance,

(3) $$\langle f_{i}\left(t\right)f_{j}\left(t^{\prime }\right)\rangle =\frac{2k_{B}T}{\mu }\;\delta_{ij}\delta \left(t-t^{\prime }\right),$$

with *T* denoting the temperature of the medium. The whole system is assumed to be at thermal equilibrium, which implies spacial homogeneity and temporal steadiness.

The interaction between the particles is expressed by the pairwise potential, V(r). We could choose any family of V(r) that interpolates between the limiting case of V=0 and the opposite limit of the hardcore potential,

(4) $$V_{\mathrm{HC}}\left(r\right)=\left\{\begin{array}{cc} \infty & \left(\left|r\right|\leq \sigma \right)\\ 0 & \left(\left|r\right|>\sigma \right) \end{array}\right.$$

with the diameter σ. Here, we choose

(5) $$V\left(r\right)=\left\{\begin{array}{cc} V_{\max}{\left(1-\frac{\left|r\right|}{\sigma }\right)}^{2} & \left(\left|r\right|\leq \sigma \right)\\ 0 & \left(\left|r\right|>\sigma \right) \end{array}\right.$$

which is parametrized by the barrier height $V_{\max}$. We also tested some other potentials [19], only to find that the basic behavior of the 1D system is qualitatively unaffected by different choices of V(r). Preference was given to Equation (5) merely because its hard sphere limit ($V_{\max}\gg k_{B}T$) has been studied systematically [31] in the context of 3D glassy dynamics.

In regard to the system governed by Equation (2), we refer to the case of $V_{\max}\to +\infty$ as the *ideal SFD*, in which every particle is eternally caged by its neighbors. Large but finite values of $V_{\max}$ allow the particle to exchange positions with one of its neighbors as a rare event which we call *overtaking* (borrowing the word from traffic flow). The ideal SFD means SFD without overtaking, and we may say “non-ideal SFD” referring to the case of finite $V_{\max}/k_{B}T$. Note that the description of non-ideal SFD with the 1D equation (2) can be interpreted as modeling a quasi-1D system [19,23,24,25,26,27] in which, typically, the position vector $r_{i}=\left(X_{i},Y_{i}\right)$ is governed by Equation (1a) with the potential term

(6) $$U=U\left(r_{1},r_{2},\ldots \right)=\sum_{\left(j,k\right)}V_{jk}+\sum_{j}V_{\mathrm{ex}}\left(Y_{j}\right),$$

where $V_{\mathrm{ex}}=V_{\mathrm{ex}}\left(y\right)$ denotes the external confinement potential such that $V_{\mathrm{ex}}\left(\pm \infty \right)\to +\infty$. In this description, $V(X_{k}-X_{j})$ in Equation (2) represents the free energy of the subsystem consisting of the neighboring particles *j* and *k*.

The specification of the system by Equations (2), (3) and (5), supplemented with the periodic boundary condition, involves some dimensional constants. As the basic scales of the length and the time, we take the particle diameter (σ) and the corresponding diffusive time ($\sigma^{2}/D$), where $D=k_{B}T/\mu$ is the diffusion constant of a free Brownian particle. A finite value of mass, such that $m/\mu :\sigma^{2}/D=1:1$, is specified for computational ease, unless specified otherwise. The system size, *L*, must be infinitely large; though, in numerical computations, we must specify some finite values for it. For later convenience, we introduce $\ell_{0}\overset{\mathrm{def}}{=}L/N=1/\rho_{0}$ which has the dimension of length. The nondimensional barrier height, $V_{\max}/k_{B}T$, has an effect on the dynamics through the overtaking frequency, as will be discussed later.

In numerical simulations, the system is equilibrated by a preparatory run started at $t=-T_{w}$. Subsequently, for a reason clarified in the next subsection, the particles are renumbered consecutively in the sense that

(7) $$X_{0}<X_{1}<\cdots <X_{i}<X_{i+1}<\cdots <X_{N}\;\left(=X_{0}+L\right)$$

at t=0. It should be noted that $T_{w}$ must be longer than maxt for sufficient equilibration [4].

### 2.2. Spatiotemporally Correlated Motion in SFD

**Figure 2:**
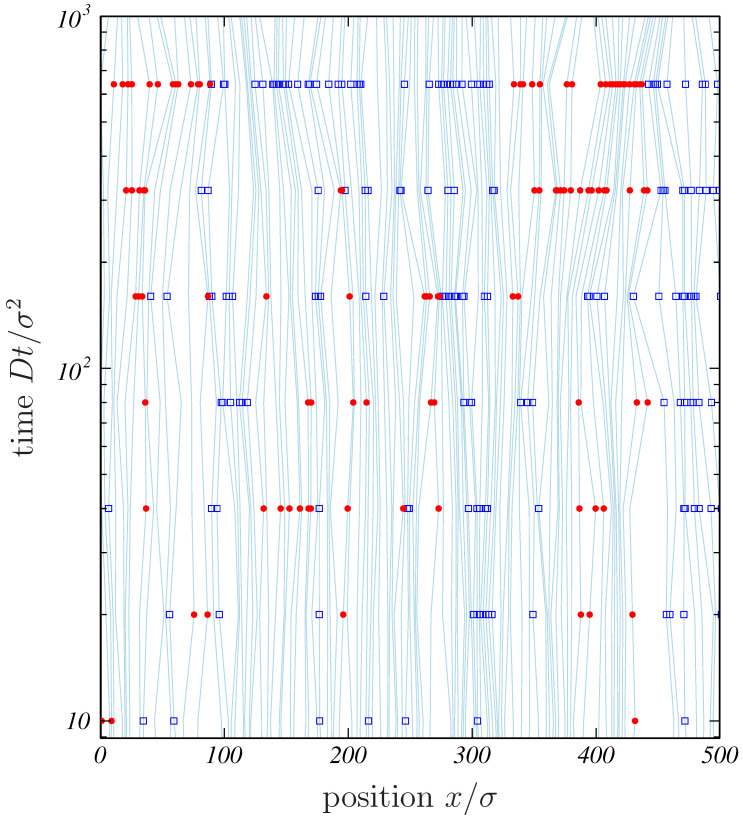
A space–time diagram representing cooperative motion in SFD. A numerical solution to Equation (2), calculated for $\rho_{0}=N/L=0.20\;\sigma^{-1}$, is plotted as worldlines in the (x,t)-plane (note that the *t*-axis is on a logarithmic scale). The symbols • and □ mark particles displaced (by more than 5σ) rightward and leftward, respectively.

As a graphic depiction of collective motions in SFD, let us examine Figure 2, in which a numerical solution of Equation (2) in the case of the ideal SFD is represented as worldlines in the (x,t)-plane. To visualize the correlation of the worldlines, we measured the displacement for each particle *i*,

(8) $$R_{i}=R_{i}\left(t\right)\overset{\mathrm{def}}{=}X_{i}\left(t\right)-X_{i}\left(0\right),$$

for the time interval from 0 to $t=2^{n}\times 10\;\sigma^{2}/D$ (with n=1,2,…), in accordance with Ref. [4]. If $R_{i}\left(t\right)>5\sigma$, the position of the particle is marked with a filled circle (•); if $R_{i}\left(t\right)<-5\sigma$, it is marked with an open square (□). As the time difference (*t*) increases, a string of the same kind of symbol is formed, expressing a cluster of particles moving together in the same direction.

The formation of clusters, visually shown in Figure 2, is quantified by calculating the displacement correlation $\langle R_{i}R_{j}\rangle$. Note that the consecutive numbering in Equation (7) is needed to make $\langle R_{i}R_{j}\rangle$ meaningful as a function of j−i(=Δ) and *t*. Since $R_{i}$ and $R_{j}$ have the same sign within the same cluster, their product must be positive if the distance in the numbering, Δ=j−i, is small, while $\langle R_{i}R_{j}\rangle$ for distant particles (with $\ell_{0}\Delta$ greater than the correlation length) is expected to vanish. The average, denoted by  , is taken over the initial condition and the Langevin noise. Computationally, the displacement correlation is calculated as

(9) $$\langle R_{i}R_{j}\rangle =\langle R_{i}R_{i+\Delta }\rangle =\frac{1}{N}\sum_{i}\langle R_{i}R_{i+\Delta }\rangle .$$

### 2.3. Continuum Description

Our theoretical approach to the displacement correlation, $\langle R_{i}R_{j}\rangle$, is based on a continuum description of the dynamics of Brownian particles. As fluctuating hydrodynamic fields describing the temporally coarse-grained dynamics of ${\left\{X_{i}\right\}}_{i=1,2,\ldots }$ for timescales longer than m/μ, one may take the density fields

(10) $$\rho \left(x,t\right)=\sum_{i}\rho_{i}\left(x,t\right),\;\rho_{i}\left(x,t\right)=\delta \left(x-X_{i}\left(t\right)\right),$$

and their fluxes

(11) $$Q\left(x,t\right)=\sum_{i}Q_{i}\left(x,t\right),\;Q_{i}\left(x,t\right)=\rho_{i}\left(x,t\right){\dot{X}}_{i}\left(t\right).$$

Note that the delta function in Equation (10) should be regarded as a blunted one, as a result of the coarse-graining (see §II-B in Ref. [21] and references therein).

The density field, ρ(x,t), is governed by the Dean–Kawasaki equation [32,33,34,35,36,37], which can be presented as a set of equations of the following form:

(12a) $$\begin{array}{c} \partial_{t}\rho +\partial_{x}Q=0 \end{array},$$

(12b) $$\begin{array}{c} Q=-D\;\left(\partial_{x}\rho +\frac{\rho }{k_{B}T}\partial_{x}U\right)+\sum_{i}\rho_{i}\left(x,t\right)f_{i}\left(t\right) \end{array},$$

(12c) $$\begin{array}{c} U=U\left[\rho \right]\left(x\right)=\int V_{\mathrm{eff}}\left(x-x^{\prime }\right)\rho \left(x^{\prime }\right)dx^{\prime } \end{array}.$$

The effective potential, $V_{\mathrm{eff}}$ in Equation (12c), is determined by the condition that the density fluctuation, described by Equations (12), should be consistent with the static structure factor,

(13) $$S\left(k\right)\overset{\mathrm{def}}{=}\frac{1}{N}\sum_{i}\sum_{j}\langle \exp\left[ik\left(X_{j}-X_{i}\right)\right]\rangle \;\left(k\neq 0\right),$$

determined directly from Equation (2). More specifically, with the Fourier mode of the density and its correlation defined as

$$\hat{\rho }\left(k,t\right)\overset{\mathrm{def}}{=}\int e^{ikx}\rho \left(x,t\right)dx=\sum_{j}\exp\left[ikX_{j}\left(t\right)\right],\;F\left(k,t\right)\overset{\mathrm{def}}{=}\frac{1}{N}\langle \hat{\rho }\left(k,t\right)\hat{\rho }\left(-k,0\right)\rangle \;\left(k\neq 0\right),$$

evidently, F(k,t=0) must be equal to S(k), and the initial decay of F(k,t) is known to be exponential, as is shown in Ref. [38] as Equation (4.144). In our notation, it reads

(14) $$F\left(k,t\right)=S\left(k\right)e^{-D^{c}k^{2}t}\;\left(\mathrm{for}\;t\ll \sigma^{2}/D\right),$$

with $D^{c}=D^{c}\left(k\right)=D/S\left(k\right)$ referred to as the (short-time) collective diffusion coefficient [38,39]. Once $V_{\mathrm{eff}}$ is determined so as to make the linearized dynamics of Equations (12) consistent with Equation (14), we may redefine S(k) by the ratio of *D* to $D^{c}\left(k\right)$.

### 2.4. Label Variable

Now let us outline the main idea that makes it possible to calculate $\langle R_{i}R_{j}\rangle$ analytically on the ground of Equations (12) [4,10,19,30]. The point is to introduce a new variable, ξ, referred to as the *label variable*, to incorporate the notion of particle tracking into the mathematical formalism that links $\langle R_{i}R_{j}\rangle$ with Equations (12).

The necessity of the new variable for particle tracking is understood by noticing how a more straightforward approach, based on $\rho_{i}\left(x,t\right)$ in Equation (10), is confronted by a difficulty. In principle, $\langle R_{i}R_{j}\rangle$ can be obtained from the Fourier mode of $\rho_{i}\left(x,t\right)$, because the definition

(15) $${\hat{\rho }}_{i}\left(k,t\right)\overset{\mathrm{def}}{=}\int e^{ikx}\rho_{i}\left(x,t\right)dx=\exp\left[ikX_{i}\left(t\right)\right]$$

implies ${\hat{\rho }}_{i}\left(k,t\right){\hat{\rho }}_{i}\left(-k,0\right)=\exp\left(ikR_{i}\right)$, and therefore

(16) $$\langle {\hat{\rho }}_{i}\left(k,t\right){\hat{\rho }}_{i}\left(-k,0\right){\hat{\rho }}_{j}\left(\pm k,t\right){\hat{\rho }}_{j}\left(\mp k,0\right)\rangle =\langle e^{ik(R_{i}\pm R_{j})}\rangle =1-\frac{k^{2}}{2}\langle {\left(R_{i}\pm R_{j}\right)}^{2}\rangle +\cdots .$$

If the correlation on the left side is successfully evaluated by nonlinear analysis of the equation governing $\rho_{i}$ and $\rho_{j}$, analogous to Equation (12b) and later shown as Equation (48), then $\langle R_{i}R_{j}\rangle$ can be obtained from the power series on the right side. This is insurmountable, unfortunately, as is evident from the complication encountered in the apparently easier problem of evaluating $\langle {\hat{\rho }}_{i}\left(k,t\right){\hat{\rho }}_{i}\left(-k,0\right)\rangle$ in SFD [8,9].

The difficulty originates from the choice of the standard field representation with the independent variables (x,t), referred to as the Eulerian description (according to the terminology of fluid mechanics [40,41]). Since $\langle R_{i}R_{j}\rangle$ is treated as a kind of *four-point* space–time correlation [42,43], it implies a *four-body* correlation in the Eulerian description, as is seen in Equation (16). However, this difficulty can be avoided by switching to another way referred to as the Lagrangian description [30,40,41], which means to include particle tracking mechanism in the definition of the fields and their correlations. In this way, the displacement correlation can be treated simply as a *two-body* Lagrangian correlation of the field [4,10,19].

Deferring a concrete calculation of $\langle R_{i}R_{j}\rangle$ until the next section, here we only define the label variable ξ to lay the foundation for it. Instead of (x,t) for the standard space–time coordinate system, we introduce a stretchable coordinate system (ξ,t), requiring ξ=ξ(x,t) to satisfy the convective equation,

(17) $$(\rho \partial_{t}+Q\partial_{x})\xi =0,$$

which states that ξ should be convected with the velocity u=Q/ρ. To satisfy Equation (17), we *define* ξ as a solution to

(18) $$\left(\rho ,Q\right)=\left(\partial_{x}\xi ,\;-\partial_{t}\xi \right).$$

This is solvable because of the continuity equation (12a), with the solution determined uniquely by some initial condition, such as $\xi (X_{0}\left(t_{0}\right),t_{0})=0$. Subsequently, by inverting the mapping (x,t)↦ξ, we obtain the coordinate system with the independent variables (ξ,t) [4,10,19,21,30]. It should be emphasized that we take Equation (18), not Equation (17), as the definition of the mapping between ξ and *x*. In other words, we do not *solve* Equation (17) in the usual sense of the word; rather, Equation (17) is satisfied as a consequence of Equation (18). In this way, we avoid complication of the attempt to define ξ=ξ(x,t) directly with Equation (17), which would require specification of the initial condition We also remind the readers that the delta function in the definition of ρ is a blunted one, as has been noted immediately after Equations (10) and (11).

Using the label variable ξ, defined in this way, we can calculate $\langle R_{i}R_{j}\rangle$ analytically. The (ξ,t) coordinate system has an advantage of making it easy to trace the worldlines, such as the ones plotted in Figure 2, because ξ is expected to keep the same value if we follow the identical particle. In order to see it, we define

(19) $$\Xi_{i}\left(t\right)\overset{\mathrm{def}}{=}\xi \left(X_{i}\left(t\right),t\right)$$

as a function of the particle number *i* and the time *t*, and consider its *t*-derivative [30,44]. Provided that the conventional chain rule is valid, the time-derivative of $\Xi_{i}\left(t\right)$ is

(20) $$\frac{d\Xi_{i}\left(t\right)}{dt}={\dot{X}}_{i}\left(t\right){\left.\frac{\partial \xi }{\partial x}\right|}_{x=X_{i}}+{\left.\frac{\partial \xi }{\partial t}\right|}_{x=X_{i}}={\left.\left(\rho {\dot{X}}_{i}-Q\right)\right|}_{x=X_{i}},$$

where the definition of ξ in Equation (18) is taken into account. The expression on the right side of Equation (20) vanishes unless the *i*-th particle overlaps with another. In the ideal SFD, in which the particles never overlap, $\Xi_{i}\left(t\right)$ is none other than the numbering *i*; this is the key ingredient for the analytical calculation of $\langle R_{i}R_{j}\rangle$ in the ideal SFD.

**Figure 3:**
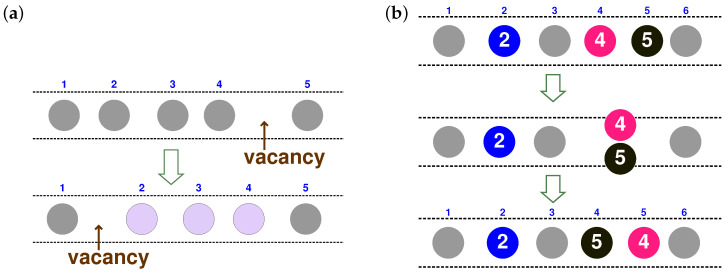
Schematic illustration of two kinds of processes in the 1D system under consideration. The small digits represent the values of the label variable ξ, while the larger digits are numbers to identify the particles. (**a**) Fluctuation of the inter-particle space without overtaking, interpretable as the migration of a vacancy. (**b**) An overtaking event, in which two particles exchange their labels.

In the absence of overtaking, the particles can move only as a result of changes in inter-particle spaces, which is illustrated schematically in Figure 3a as a migrating “vacancy” causing correlated motion of particles. The overtaking allows another kind of motion, illustrated in Figure 3b, which does not require migration of a vacancy. Before proceeding to a concrete calculation of $\langle R_{i}R_{j}\rangle$, let us discuss how to describe an overtaking event within the framework of the (ξ,t) coordinate system.

### 2.5. Kinematics of Overtaking Events

In the presence of overtaking, $\Xi_{i}\left(t\right)=\xi \left(X_{i}\left(t\right),t\right)$ is not necessarily equal to *i*. In this case, we must distinguish between them and discuss correspondence among three variables: the numbering (i∈Z), the label variable (ξ∈R), and the position (*x*).

We still define ξ by Equation (18) without regard to overtaking. The constant of integration is chosen appropriately so that $\Xi_{i}\left(t\right)=\xi \left(X_{i}\left(t\right),t\right)$ has an integer value unless the *i*-th particle overlaps with another. On this premise, the mapping from i∈Z to $\Xi_{i}\left(t\right)=\xi \left(X_{i}\left(t\right),t\right)\in Z$ is injective, which means, so to speak, a kind of exclusion principle whereby different particles must carry different values of ξ.

The overtaking process is a transition from one such mapping to another. Since the effect of overtaking on the mapping $i\mapsto \Xi_{i}\left(t\right)$ is local, as is readily seen from Equation (18), we may identify the overtaking event with a process in which two particles exchange the value of their label. In the case of a pair of particles (i,j), with $t_{1}$ and $t_{2}$ denoting points in time immediately before and after the exchange, this process is described as

(21) $$\Xi_{i}\left(t_{2}\right)=\Xi_{j}\left(t_{1}\right),\;\Xi_{j}\left(t_{2}\right)=\Xi_{i}\left(t_{1}\right).$$

To justify Equation (21), we consider what occurs in the time interval from $t_{1}$ to $t_{2}$. Since the particles may overlap in the course of overtaking, the value of $\Xi_{i}\left(t\right)$ is not limited to Z but rather belongs to R. Using ρ and $\rho_{i}$ given in Equation (10), we rewrite Equation (20) as

(22) $$\begin{array}{cc} \frac{d\Xi_{i}}{dt} & =\int \left(\rho {\dot{X}}_{i}-Q\right)\rho_{i}dx=\sum_{j}\int \left(\rho_{j}{\dot{X}}_{i}-Q_{j}\right)\rho_{i}dx\\ & =\sum_{j}\int \left(\rho_{j}Q_{i}-\rho_{i}Q_{j}\right)dx, \end{array}$$

which implies that $\Xi_{i}\left(t\right)$ is locally conserved. Note that only overlapping particles contribute to the integral. If no particle overlaps the *i*-th one, then the expression on the right side of Equation (20) vanishes so that $\Xi_{i}$ remains constant. If only one particle, say the *j*-th one, overlaps particle *i*, Equation (22) gives

(23) $$\frac{d\Xi_{i}}{dt}=\int \left(\rho_{j}Q_{i}-\rho_{i}Q_{j}\right)dx=-\frac{d\Xi_{j}}{dt}.$$

By integrating Equation (23) over the time interval from $t_{1}$ to $t_{2}$ and taking the conditions such as $\Xi_{i}\left(t_{1}\right)\in Z$ into account, we find

$$\Xi_{i}\left(t_{2}\right)-\Xi_{i}\left(t_{1}\right)=-\Xi_{j}\left(t_{2}\right)+\Xi_{j}\left(t_{1}\right)\in Z,$$

so that only two cases are possible: one is the case of unsuccessful or retracted overtaking, with the same value of $\left(\Xi_{i},\Xi_{j}\right)$ restored after the interaction, and the other case corresponds to Equation (21) that describes (successful) overtaking. Note that the other possibilities are eliminated by the “exclusion principle” for the configurations before and after the overtaking process. As a rare case, three-body or four-body interactions might be possible, but it suffices to approximate such a case with a sequence of two-body exchange processes.

Changes of $\Xi_{i}\left(t\right)=\xi \left(X_{i}\left(t\right),t\right)$ and $X_{i}\left(t\right)=x\left(\Xi_{i}\left(t\right),t\right)$ are exemplified in Figure 3. Since ξ=ξ(x,t) satisfies ∂ξ/∂x=ρ by definition in Equation (18), $\left\{\Xi_{i}\right\}$ is always spatially consecutive, as is shown in Figure 3 with small digits. The important point is that this spatial consecutiveness of $\left\{\Xi_{i}\right\}$ holds true even if the numbering is inverted by overtaking. In other words, ξ labels the order in the file and not the particles themselves. In the case of Figure 3b, the particles are initially numbered consecutively so that $\Xi_{i}=i$ for all *i*, until particles 4 and 5 start to overlap. During overtaking, the values of $\Xi_{4}$ and $\Xi_{5}$ evolve according to Equation (23) with (i,j)=(4,5), while the labels for other particles, such as $\Xi_{2}$, $\Xi_{3}$ and $\Xi_{6}$, remain unchanged. Finally, after overtaking, particle 4 carries the label $\Xi_{4}=5$, while label 4 is now carried by particle 5 so that $\Xi_{5}=4$.

In the next section, we start with the case of the ideal SFD, in which the requirement of $\left(d/dt\right)\Xi_{i}\left(t\right)=0$ is fulfilled so that ξ simply plays the role of particle numbering. Later, we consider the temporal change of $\Xi_{i}\left(t\right)$ to allow for overtaking in Section 4.

## 3. Displacement Correlation in SFD without Overtaking

### 3.1. Analytical Calculation of Displacement Correlation

Here we review the analytical calculation of $\langle R_{i}R_{j}\rangle$ in the ideal SFD [4,10,19], in which $\Xi_{i}$ is independent of *t*.

Out of the three equations composing the Dean–Kawasaki Equation (12), the continuity Equation (12a) is already included in ξ=ξ(x,t), or its inverse mapping x=x(ξ,t), through Equation (18). To relate x=x(ξ,t) to the remaining two equations, we solve Equation (12b) for u=Q/ρ, which should be equal to $u=\partial_{t}x(\xi ,t)$. Noticing that u(ξ,t) is the (negative) flux of $1/\rho =\partial_{\xi }x(\xi ,t)$, we introduce [30]

(24) $$\psi =\psi \left(\xi ,t\right)\overset{\mathrm{def}}{=}\rho_{0}\frac{\partial x}{\partial \xi }-1$$

so that $1/\rho =\ell_{0}\left(1+\psi \right)$. The fluctuating field ψ can be interpreted as a continuum representation of migrating vacancies [16] or elongation of a chain [45]. As is illustrated schematically in Figure 3a, migration of a vacancy can give rise to correlated motion of particles.

Once the dynamics of ψ are known from Equations (12), the remaining task for the calculation of $\langle R_{i}R_{j}\rangle$ is only a problem of kinematics—given the field ψ, how can we find the displacement? This is readily solved in a Fourier representation,

(25) $$\psi \left(\xi ,t\right)=\sum_{k}\overset{ˇ}{\psi }\left(k,t\right)e^{-ik\xi },\;\overset{ˇ}{\psi }\left(k,t\right)=\int e^{ik\xi }\psi \left(\xi ,t\right)\frac{d\xi }{N}\;\left(\mathrm{with}\;\;\frac{k}{2\pi /N}\in Z\right),$$

allowing us to express x=x(ξ,t) as an antiderivative of ψ:

(26) $$x=x\left(\xi ,t\right)=\ell_{0}\xi +\ell_{0}\sum_{k}e^{-ik\xi }\frac{\overset{ˇ}{\psi }\left(k,t\right)}{-ik}+X_{G}\left(t\right),$$

where $X_{G}\left(t\right)$ corresponds to the center-of-mass motion which should be negligible in the limit of N→+∞ [10]. The positions of the *i*-th or *j*-th particle are then obtained by substituting $\Xi_{i}$ or $\Xi_{j}$ into ξ in Equation (26), which readily yields

(27) $$R_{i}=x\left(\Xi_{i},t\right)-x\left(\Xi_{i},0\right)=\ell_{0}\sum_{k}e^{-ik\Xi_{i}}\frac{\overset{ˇ}{\psi }\left(k,t\right)-\overset{ˇ}{\psi }\left(k,0\right)}{-ik}$$

in the absence of overtaking (i.e., $d\Xi_{i}/dt=0$). To calculate $\langle R_{i}R_{j}\rangle$, we multiply Equation (27) by its duplicate with (i,k) replaced by (j,−k). Subsequently, taking it into account that $\langle \overset{ˇ}{\psi }\left(k,t\right)\overset{ˇ}{\psi }\left(k^{\prime },t^{\prime }\right)\rangle$ generally vanishes unless $k+k^{\prime }=0$ (due to the space-translation symmetry of the system), we find

(28) $$\begin{array}{cc} \langle R_{i}R_{j}\rangle & =\ell_{0}^{2}\sum_{k}\frac{e^{-ik(\Xi_{j}-\Xi_{i})}\langle \left[\overset{ˇ}{\psi }\left(k,t\right)-\overset{ˇ}{\psi }\left(k,0\right)\right]\left[\overset{ˇ}{\psi }\left(-k,t\right)-\overset{ˇ}{\psi }\left(-k,0\right)\right]\rangle }{k^{2}}\\ & \to \frac{\ell_{0}^{2}}{\pi }\int_{-\infty }^{\infty }e^{-ik\Delta }\frac{C_{\psi }\left(k,0\right)-C_{\psi }\left(k,t\right)}{k^{2}}dk\;\left(N\to \infty \right), \end{array}$$

where we have defined

(29) $$C_{\psi }\left(k,t\right)\overset{\mathrm{def}}{=}N\langle \overset{ˇ}{\psi }\left(k,t\right)\overset{ˇ}{\psi }\left(-k,0\right)\rangle$$

and used $\Xi_{j}-\Xi_{i}=j-i=\Delta$. We refer to Equation (28) as the Alexander–Pincus formula [10,12], which relates the displacement correlation to $C_{\psi }$. Since $C_{\psi }$ is a *two-body* correlation, it is much more tractable than the four-body correlation in Equation (16).

To allow concrete calculation of $C_{\psi }$, Equations (12b) and (12c) are rewritten as an equation for $\overset{ˇ}{\psi }\left(k,t\right)$ in the following form:

(30) $$\partial_{t}\overset{ˇ}{\psi }\left(k,t\right)=-D_{*}^{c}k^{2}\overset{ˇ}{\psi }\left(k,t\right)+\sum_{p+q+k=0}V_{k}^{pq}\overset{ˇ}{\psi }\left(-p,t\right)\overset{ˇ}{\psi }\left(-q,t\right)+O\left({\overset{ˇ}{\psi }}^{3}\right)+\rho_{0}{\overset{ˇ}{f}}_{L}\left(k,t\right).$$

The statistics of the random force term are specified as

(31) $$\rho_{0}^{2}\langle {\overset{ˇ}{f}}_{L}\left(k,t\right){\overset{ˇ}{f}}_{L}\left(-k^{\prime },t^{\prime }\right)\rangle =\frac{2D_{*}}{N}k^{2}\delta_{kk^{\prime }}\delta \left(t-t^{\prime }\right),$$

where $D_{*}=D/\ell_{0}^{2}$. The coefficient of the linear term is

(32) $$D_{*}^{c}=\frac{D^{c}}{\ell_{0}^{2}}=D_{*}\left(1+\frac{2\sin\rho_{0}\sigma k}{k}\right),$$

which also gives $S=D/D^{c}=D_{*}/D_{*}^{c}$, and the coefficients $V_{k}^{pq}$ in the nonlinear term are found in Refs. [4,30].

Within the linear approximation to Equation (30), $C_{\psi }$ is readily calculated as

(33) $$C_{\psi }\left(k,t\right)=Se^{-D_{*}^{c}k^{2}t}.$$

Although a nonlinear theory producing a correction term to be added to Equation (33) is also possible [4], here we ignore this correction, giving priority to the simpler expression in Equation (33).

The displacement correlation is obtained by substituting Equation (33) into the Alexander–Pincus Formula (28) and evaluating the integral. As the contribution from the longwave modes is dominant, S=S(k) and $D_{*}^{c}=D_{*}/S\left(k\right)$ can be replaced by their limiting values for k→+0. Thus, $\langle R_{i}R_{j}\rangle$ is obtained explicitly as a function of Δ and *t*, expressible in terms of a similarity variable

(34) $$\theta \overset{\mathrm{def}}{=}\frac{\ell_{0}\Delta }{\lambda (t)}=\frac{\Delta }{2\sqrt{D_{*}^{c}t}},\;\lambda \left(t\right)=2\sqrt{D^{c}t}\;,$$

as [4,10,19]

(35) $$\frac{\langle R_{i}R_{j}\rangle }{2S\ell_{0}^{2}\sqrt{(D_{*}^{c}/\pi )t}}=e^{-\theta^{2}}-\sqrt{\pi }\left|\theta \right|\mathrm{erfc}\left|\theta \right|\overset{\mathrm{def}}{=}\varphi \left(\theta \right),$$

so that $\langle R_{i}R_{j}\rangle =K\sqrt{t}\;\varphi \left(\theta \right)$ where $K=2S\ell_{0}^{2}\sqrt{D_{*}^{c}/\pi }$. The dynamical correlation length λ(t) grows diffusively, which seems to be consistent with the observation of growing clusters in Figure 2.

Note that infrared and ultraviolet cutoffs are *not* necessary in Equation (28), as the integrand is regular around k=0 and decays for k→±∞ (algebraically, but fast enough). This should not be confused with the infrared divergence of x(ξ,t) in Equation (26) in the limit of L→∞.

### 3.2. Particle Simulation in the Absence of Overtaking

**Figure 4:**
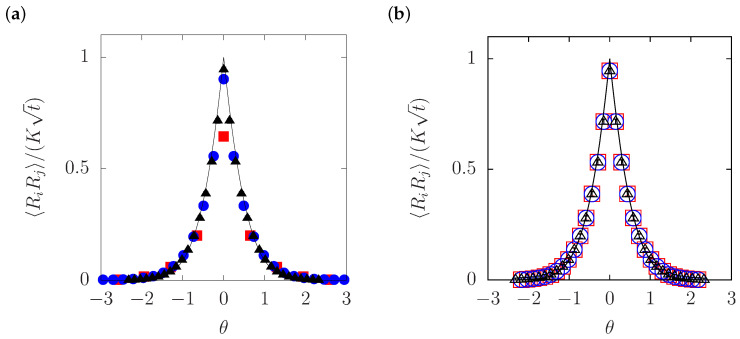
Comparison of the theoretical prediction (35) for the ideal single-file diffusion (SFD) with a particle-based simulation. (**a**) Displacement correlation for different values of the time interval *t*. The red squares (▪), the blue circles (•) and the black triangles (▴) represent the numerical values of $\langle R_{i}R_{j}\rangle /\left(K\sqrt{t}\;\right)$ at the time intervals $t/(\sigma^{2}/D)=10$, 70 and 200, respectively. The ratio m/μ:$\sigma^{2}/D$ was chosen to be 1:1. The values are plotted against $\theta =\ell_{0}\Delta /\lambda \left(t\right)$, i.e., the distance rescaled with λ(t). The thin, solid line shows the theoretical master curve given by φ(·) in Equation (35). (**b**) Displacement correlation for different values of m/μ. The open squares (□) represent the numerical result for m/μ:$\sigma^{2}/D$ = 1:1, the open circles (∘) for 1:2, and the open triangles (▵) for 1:5. The time interval was chosen to be $t=200\;\sigma^{2}/D$.

The theoretical prediction in Equation (35) is tested in Figure 4a, by plotting the values of the displacement correlation, $\langle R_{i}R_{j}\rangle$, obtained from numerical simulation of the ideal SFD. The computed values are plotted in terms of rescaled variables; according to Equation (35), a plot of $\langle R_{i}R_{j}\rangle /\left(K\sqrt{t}\right)$ against the similarity variable θ should give a single master curve for all values of the time interval *t*. The ideal SFD was simulated by solving Equation (2) numerically with a very high barrier (we chose $V_{\max}=50k_{B}T$). The system size and the density were specified as $N=10^{4}$ and $\rho_{0}=N/L=0.2\;\sigma^{-1}$ so that L=5Nσ.

The three kinds of symbols in Figure 4a correspond to three different values of *t*. The plots for all these values of *t* are seen to be reducible to a single master curve given by φ(·) in Equation (35), supporting the prediction of the analytical calculation.

To be precise, a small but finite discrepancy is found at θ=0 for $t=10\;\sigma^{2}/D$. One may be tempted to explain this short-time discrepancy simply as indicating a lack of time to establish correlated motion, because a particle, on average, takes time on the order of $1/D_{*}^{c}$ to encounter its neighbors. Unfortunately, this argument appears too simple to explain the numerical result in Figure 4a in which a positive correlation for θ≠0 (i.e., $\left|\Delta \right|\geq 1$) has already been established at $t=10\;\sigma^{2}/D$. We note, on the other hand, that the discrepancy can be ascribed to the nonlinear term in Equation (30) which was ignored in the previous subsection. It is shown that inclusion of the nonlinear term gives a correction to Equation (35), which is significant only for $\left|\theta \right|\ll 1$ and $D_{*}^{c}t<1$ [4], and the sign of the correction for the MSD is negative [4,10]. Thus, the short-distance correlations are affected by nonlinear coupling of $\overset{ˇ}{\psi }$, while correlations over a long distance seem to be tractable with a linear theory.

It should be also noted that the theoretical predictions discussed here are based on the Dean–Kawasaki equation in which inertia is completely ignored, while the particle-based simulation is performed with finite m/μ. To check for consistency between the numerical simulation and the theoretical predictions, three cases with different values of m/μ are compared in Figure 4b. It is seen that the change in m/μ does not make any remarkable difference. This is in agreement with the general expectation that the Langevin dynamics on time scales longer than m/μ are basically independent of the inertia, because the momentum can be eliminated by temporal coarse-graining [46,47,48].

In regard to correlations over long distances, one might be tempted to suppose that the static structure factor, S(k) in Equation (13), could be helpful in the detection of such long-ranged correlations. This point was discussed in Ref. [19], leading to the conclusion that the structure of the collective motion is not properly captured by the static structure factor. It is for this reason that we focused on $\langle R_{i}R_{j}\rangle$ rather than S(k).

## 4. Effects of Overtaking on Displacement Correlation

Having reviewed the analytical calculation of the displacement correlation $\langle R_{i}R_{j}\rangle$, which leads to Equation (35) for the ideal SFD, let us now consider effects of overtaking that were ignored in the previous section. We start with numerical observation, noticing how the behavior of $\langle R_{i}R_{j}\rangle$ deviates from Equation (35) due to overtaking. This deviation is then compared with a modified theory in which overtaking is allowed for.

### 4.1. Particle Simulation of SFD with Overtaking

The governing equations of the system, namely Equations (2), (3) and (5), contain a parameter $V_{\max}$ representing the barrier height. This parameter, $V_{\max}$, regulates the frequency of overtaking, if the other parameters are kept unchanged.

Two extreme cases are already known theoretically: the case of $V_{\max}\to \infty$ implying the ideal SFD in which overtaking is completely forbidden, and $V_{\max}=0$ corresponding to free diffusion in which overtaking is always allowed. In the ideal SFD, there is a positive correlation between displacements of two particles, as is shown in Equation (35), while in free diffusion, displacements of two particles are totally uncorrelated, as is easily seen by proving

(36) $$\langle R_{i}R_{j}\rangle =\left\{\begin{array}{cc} 2Dt+O(Dm/\mu ) & \left(i=j\right)\\ 0 & \left(i\neq j\right) \end{array}\right.$$

for $V_{\max}=0$.

**Figure 5:**
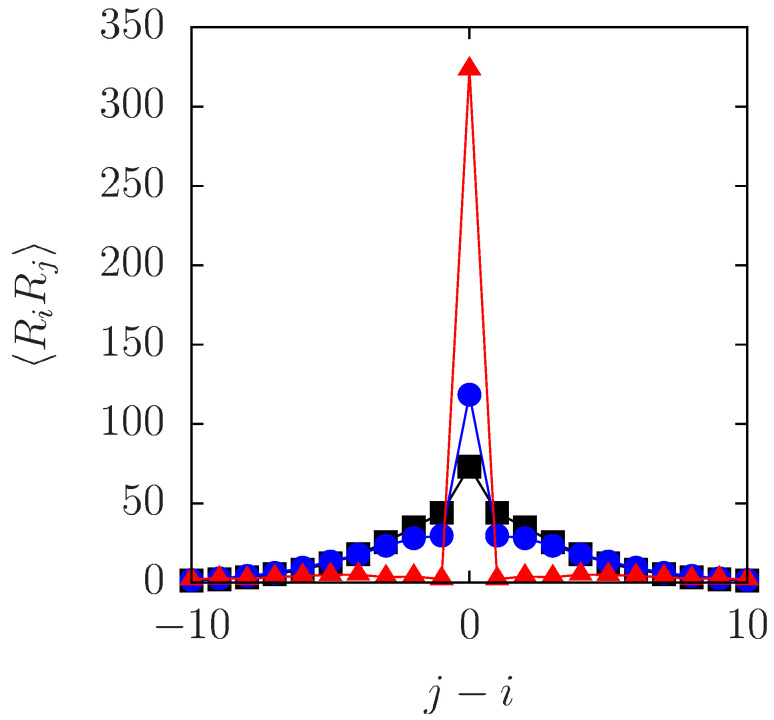
Two-particle displacement correlation, $\langle R_{i}R_{j}\rangle$ (nondimensionalized with $\sigma^{2}$), numerically obtained for three different values of $V_{\max}$. The red triangles (▴), the blue circles (•) and the black squares (*▪*) represent the data for $V_{\max}=k_{B}T$, $3k_{B}T$ and $5k_{B}T$, respectively. The time interval is fixed at $t=200\sigma^{2}/D$. The density and the system size are the same as in Figure 4.

Between these two extreme cases, there are cases of finite values of $V_{\max}$, allowing overtaking with some probability. Three such cases are shown in Figure 5, where the computed values of $\langle R_{i}R_{j}\rangle$ at $t=200\sigma^{2}/D$ are plotted against Δ=j−i. In the case of the lowest barrier, $V_{\max}=k_{B}T$, the plot is similar to Equation (36) in that $\langle R_{i}R_{j}\rangle$ almost vanishes for i≠j; instead, the MSD (i=j) is greater than in the other two cases, indicating that the particles are diffusing almost freely. The case of the highest barrier with $V_{\max}=5k_{B}T$ resembles the ideal SFD, although a close inspection reveals a slight deviation from Equation (35) as a result of overtaking that occurs at a very small rate.

The intermediate case with $V_{\max}=3k_{B}T$ is interesting. At large distances, the same correlation is observed in the case of $V_{\max}=3k_{B}T$ as in the case of $V_{\max}=5k_{B}T$ (and as in the ideal SFD). In contrast, at Δ=±1 and ±2, the correlation in the case of $V_{\max}=3k_{B}T$ is remarkably smaller than that for $V_{\max}=5k_{B}T$. The decrease in the displacement correlation and the increase in MSD must be attributed to overtaking.

The numerical observation on the effects of a finite $V_{\max}$ may be understood intuitively, if $\langle R_{i}R_{j}\rangle$ is regarded as representing a nested structure of cages with different radii. From this point of view, the plot for $V_{\max}=3k_{B}T$ in Figure 5 can be interpreted as describing the breakdown of inner cages, while the outer ones persist (at least until $t=200\sigma^{2}/D$). To elevate this pictorial idea to a quantitative theory on collective motion in SFD with overtaking, we raise the question: How can we modify Equation (35) allowing for overtaking?

### 4.2. Theory of Displacement Correlation in SFD with Overtaking

To find out how Equation (35) should be modified by overtaking, let us re-examine its derivation process. A crucial step is found in Equation (27) where $R_{i}$ is obtained on the assumption that $\Xi_{i}$ is independent of *t*. This is the point at which the “no overtaking” rule was enforced [30,44].

Overtaking is incorporated into the theory though temporal changes of $\Xi_{i}\left(t\right)$ [44]. The kinematics of overtaking were discussed in SubSection 2.5: as is illustrated in Figure 3, two particles exchange their labels according to Equation (21).

In order to recalculate the displacement correlation, now we need to consider the dynamics of overtaking. In principle, the stochastic dynamics of $\Xi_{i}\left(t\right)$ should be determined from “first principles” through Equation (22). As a crude approximation, however, here we adopt a phenomenological description characterized by the parameter $\nu_{\alpha }$, referred to as the *overtaking frequency* or the *hopping rate*.

The dynamics of $\Xi_{i}\left(t\right)$ is modeled as a simple Markovian process in which the labels of neighboring particles are exchanged at a rate of $\nu_{\alpha }$. More precisely speaking, for every pair (i,j) such that $\Xi_{j}\left(t_{1}\right)=\Xi_{i}\left(t_{1}\right)\pm 1$, their labels are exchanged according to Equation (21) with a probability of $1-\nu_{\alpha }\Delta t$ in every short time interval, $\Delta t=t_{2}-t_{1}$. This process is essentially equivalent to what is known as “Amida-kuji” (Amitabha’s lottery) or the “ladder lottery” [49,50]. In regard to the dynamics of a single tagged particle, the Amida-kuji process is merely a diffusion on a 1D lattice [49,51] such that

(37) $${\left[\Xi_{i}\left(t\right)-\Xi_{i}\left(0\right)\right]}^{2}=2\nu_{\alpha }t.$$

This is readily shown by solving the master equation for the one-tag diffusion propagator [51],

(38) $$\partial_{t}P(a,t)=\nu_{\alpha }\left[P(a+1,t)+P(a-1,t)-2P(a,t)\right],$$

where P(a,t)=P(0,0;a,t) denotes the probability that the tagged particle, the 0-th one such that $\Xi_{0}\left(0\right)=0$, carries the label $\Xi_{0}\left(t\right)=a$ at the time *t*.

In order to calculate the contribution of overtaking to the displacement correlation $\langle R_{i}R_{j}\rangle$, we need to know the two-body diffusion propagator, P(i,j,0;a,b,t), which represents the probability that the tagged particles, initially labelled with $\Xi_{i}\left(0\right)=i$ and $\Xi_{j}\left(0\right)=j$, are found to carry $\Xi_{i}\left(t\right)=a$ and $\Xi_{j}\left(t\right)=b$ at the time *t*. Assuming j−i=Δ>0 without loss of generality, we write the master equation for $P_{a,b}=P\left(i,i+\Delta ,0;a,b,t\right)$ with a≠b as

(39) $$\partial_{t}P_{a,b}=\nu_{\alpha }\left(P_{a+1,b}+P_{a-1,b}+P_{a,b+1}+P_{a,b-1}-4P_{a,b}\right)+\left(\delta_{a+1,b}+\delta_{a,b+1}\right)\nu_{\alpha }\left(P_{b,a}+P_{a,b}\right)$$

and $P_{a,a}=0$. This is more complicated than Equation (38) but still solvable in a Fourier representation (as is shown in Appendix A), so that two-body correlations of $\Xi_{i}\left(t\right)$ are given in terms of the modified Bessel function in the limit of N→∞.

Now, we are prepared for calculation of $\langle R_{i}R_{j}\rangle$. From Equation (26) and $R_{i}=x\left(\Xi_{i},t\right)-x\left(\Xi_{i},0\right)$, we find

(40) $$\begin{array}{cc} R_{i} & =\ell_{0}\sum_{k\neq 0}\frac{e^{-ik\Xi_{j}\left(t\right)}\overset{ˇ}{\psi }\left(k,t\right)-e^{-ik\Xi_{j}\left(0\right)}\overset{ˇ}{\psi }\left(k,0\right)}{-ik}+\ell_{0}\left[\Xi_{i}\left(t\right)-\Xi_{i}\left(0\right)\right]\\ & =\ell_{0}\sum_{k\neq 0}\frac{e^{-ik\Xi_{i}^{0}}}{-ik}\left[e^{-ik\delta \Xi_{i}}\overset{ˇ}{\psi }\left(k,t\right)-\overset{ˇ}{\psi }\left(k,0\right)\right]+\ell_{0}\delta \Xi_{i}, \end{array}$$

where we have defined

$$\Xi_{i}^{0}\overset{\mathrm{def}}{=}\Xi_{i}\left(0\right),\;\delta \Xi_{i}=\delta \Xi_{i}\left(t\right)\overset{\mathrm{def}}{=}\Xi_{i}\left(t\right)-\Xi_{i}^{0}$$

for the sake of brevity. Following the same line of argument as in the derivation of Equation (28), on the assumption that $\overset{ˇ}{\psi }$ and $\Xi_{i}$ are uncorrelated, we have

(41) $$\begin{array}{cc} \langle R_{i}R_{j}\rangle & =\ell_{0}^{2}\sum_{k\neq 0}\frac{e^{ik(\Xi_{j}^{0}-\Xi_{i}^{0})}}{k^{2}}\langle \left[e^{-ik\delta \Xi_{i}\left(t\right)}\overset{ˇ}{\psi }\left(k,t\right)-\overset{ˇ}{\psi }\left(k,0\right)\right]\left[e^{ik\delta \Xi_{j}\left(t\right)}\overset{ˇ}{\psi }\left(-k,t\right)-\overset{ˇ}{\psi }\left(-k,0\right)\right]\rangle \\ & \;+\ell_{0}^{2}\langle \delta \Xi_{i}\delta \Xi_{j}\rangle . \end{array}$$

The summand can be expanded as

(42) e−ikδΞiψˇ(k,t)−ψˇ(k,0)eikδΞjψˇ(−k,t)−ψˇ(−k,0)=eik(δΞj−δΞi)ψˇ(k,t)ψˇ(−k,t)−e−ikδΞiψˇ(k,t)ψˇ(−k,0)−eikδΞjψˇ(−k,t)ψˇ(k,0)+ψˇ(k,0)ψˇ(−k,0)≃1+eik(δΞj−δΞi)ψˇ(k,t)ψˇ(−k,t)−2Ree−ikδΞiψˇ(k,t)ψˇ(−k,0),

again, with the assumption that $\overset{ˇ}{\psi }$ and $\Xi_{i}$ are uncorrelated. The terms including the exponentials of $\delta \Xi_{i}$ can be evaluated analytically on the basis of solutions to the master equations (38) and (39).

In this way, after some calculation, we obtain the displacement correlation. For i=j, we have

(43) $$\frac{\langle {R_{i}}^{2}\rangle }{\ell_{0}^{2}}=2S\sqrt{\frac{D_{*}^{\prime }t}{\pi }}+2\nu_{\alpha }t,$$

where $D_{*}^{\prime }=D_{*}^{c}+\nu_{\alpha }$. Note that the last term, $2\nu_{\alpha }t$, originates from Equation (37). In the case of i≠j, using φ(·) defined in Equation (35), we obtain

(44) $$\frac{\langle R_{i}R_{j}\rangle }{\ell_{0}^{2}}=S\left[2\sqrt{\frac{D_{*}^{\prime }t}{\pi }}\;\varphi \;\left(\frac{\left|j-i\right|}{\sqrt{4D_{*}^{\prime }t}}\right)-\sqrt{\frac{2\nu_{\alpha }t}{\pi }}\;\varphi \;\left(\frac{\left|j-i\right|}{\sqrt{8\nu_{\alpha }t}}\right)\right]+\langle \delta \Xi_{i}\delta \Xi_{j}\rangle .$$

The last term (Δ=j−i≥1 without loss of generality) needs to be evaluated with the two-body diffusion propagator in the form of Equation (A13) in Appendix A, which yields

(45) $$\langle \delta \Xi_{i}\delta \Xi_{i+\Delta }\rangle =-2\nu_{\alpha }t\;e^{-4\nu_{\alpha }t}\left[I_{\Delta -1}\left(4\nu_{\alpha }t\right)+I_{\Delta }\left(4\nu_{\alpha }t\right)\right]+\left(\Delta -\frac{1}{2}\right)e^{-4\nu_{\alpha }t}\sum_{n=\Delta }^{\infty }I_{n}\left(4\nu_{\alpha }t\right),$$

with $I_{n}\left(\;\cdot \;\right)$ denoting the *n*-th modified Bessel function. It is easy to verify that, in the limit of $\nu_{\alpha }\to 0$, Equations (43) and (44) are reduced to the ideal case in Equation (35).

**Figure 6:**
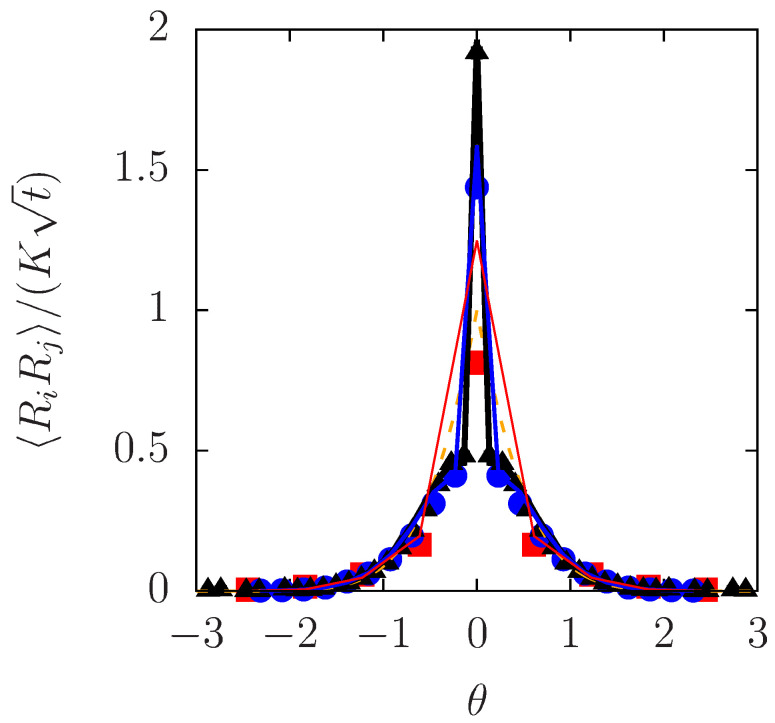
Comparison of the theoretical predictions of $\langle R_{i}R_{j}\rangle$ with particle-based numerical calculations of SFD with overtaking ($V_{\max}=3k_{B}T$). The three symbols have the same meaning as in Figure 4 with the axes rescaled also in the same way: $\langle R_{i}R_{j}\rangle /\left(K\sqrt{t}\;\right)$ is plotted against θ. The thinnest red line, the thin blue line, and the solid black line represent Equation (44) that allows for overtaking ($\nu_{\alpha }=0.0057\;D/\sigma^{2}$), evaluated at the values of *t* corresponding to the three kinds of symbols, namely, at $t/(\sigma^{2}/D)=10$, 70, and 200, respectively. The theory without overtaking [$\nu_{\alpha }=0$, i.e., Equation (35)] is shown by a broken line.

The theoretical prediction in Equations (43)–(45) is compared with a result of our particle simulation in Figure 6. With the barrier height and the density chosen as $V_{\max}=3k_{B}T$ and $\rho_{0}=N/L=0.2\;\sigma^{-1}$ ($N=10^{4}$ and L=5Nσ), we calculated $\langle R_{i}R_{j}\rangle$ and delineated it for three different values of *t*. The same rescaled variables were used as in Figure 4a: namely, $\langle R_{i}R_{j}\rangle /\left(K\sqrt{t}\right)$ is plotted against θ, with *K* given immediately below Equation (35). The hopping rate was evaluated numerically and estimated to be $\nu_{\alpha }=0.0057\;D/\sigma^{2}$ (see Appendix B), which was used to plot Equations (43)–(45) as theoretical curves in Figure 6. The prediction for the ideal SFD ($\nu_{\alpha }=0$) in Equation (35) is also included with a broken line.

In regard to the difference between the particle simulation and theory for the ideal SFD, Figure 6 exhibits qualitatively the same behavior as was observed in Figure 5—the difference due to overtaking is remarkable only for small θ and occurs in such a way that, except for the self part (i=j, i.e., the MSD), the numerical values of the displacement correlation are smaller than the prediction for the ideal SFD in Equation (35). This means that the effect of overtaking should manifest itself as a negative correction to $\langle R_{i}R_{j}\rangle$ for i≠j. In this sense, the present theory modifies Equation (35) in the right direction, as the theoretical curve in Figure 6 predicts *smaller* values of $\langle R_{i}R_{j}\rangle$ for i≠j in comparison to Equation (35).

## 5. Discussion and Concluding Remarks

We have presented calculations of two-particle displacement correlation, $\langle R_{i}R_{j}\rangle$, in a 1D or quasi-1D system of Brownian particles with repulsive interaction, i.e., in SFD with or without overtaking, as an illustrative model of collective dynamics associated with the cage effect. In the ideal SFD (without overtaking), correlated motion with a diffusively growing length scale, $\lambda =2\sqrt{D^{c}t}$, was observed. Subsequently, we studied how this result is modified by overtaking; it was shown both numerically and analytically that the overtaking processes destroy short-range correlations alone, leaving long-range correlations nearly intact. This behavior of $\langle R_{i}R_{j}\rangle$, evidenced in Figure 5 and Figure 6, suggests a spatiotemporally nested structure of cages, such that smaller cages are enclosed in larger cages with longer lifetimes.

The main objective of the present work was to shed theoretical light on overtaking in SFD, by extending an analytical theory of SFD to the case of non-ideal SFD in which overtaking is allowed. The analytical theory is based on the method of the label variable ξ. The Lagrangian correlation of the field ψ links $\langle R_{i}R_{j}\rangle$ to the Dean–Kawasaki Equation (12) that describes the fluctuating density field, while overtaking is taken into account through $\delta \Xi_{i}\left(t\right)$. The main analytical result is represented by Equations (43) and (44). This result is reasonably consistent with the numerical behavior of $\langle R_{i}R_{j}\rangle$ in Figure 6. A linear solution to the transformed Dean–Kawasaki equation (30) seems to suffice for the description of the outer cages (long scales). Contrastively, the inner cages are not only affected by overtaking via $\delta \Xi_{i}\left(t\right)$ but also subject to nonlinear coupling of $\overset{ˇ}{\psi }$, as is suggested by the deviation from the linear theory in Figure 4a.

In spite of the reasonable agreement between the analytical and numerical results, however, there are at least two issues that need to be discussed and probably improved in the future. Firstly, the hopping rate ($\nu_{\alpha }$) could be obtained from $\beta V_{\max}$ and other parameters in a more first-principle-oriented manner, as opposed to the numerical fitting adopted here. Secondly, the validity of the decoupling approximation in Equation (42) is questionable.

In regard to the first issue, we note that the numerical fit for $\nu_{\alpha }$ in Equation (A14) in Appendix B is not simply given by an Arrhenius-like expression, $e^{-\beta V_{\max}}$ [46], but includes a prefactor that depends on $\rho_{0}$ in a nontrivial way. Although accurate computation of overtaking is difficult and may require improvement in the numerical scheme, which is outside the scope of the present work, the numerical prefactor is interesting enough to motivate theoretical attempts to explain it. While $V_{\max}$ in Equation (2) corresponds to the Helmholtz free energy barrier in quasi-1D systems governed by Equations (1a) and (6) [10,27], the hopping rate is rather related to a barrier in the Gibbs free energy [52]. The $\rho_{0}$-dependent prefactor is also reminiscent of the escape probability in predator–prey problems on a 2D lattice [53]. Theoretical evaluation of the hopping rate ($\nu_{\alpha }$) in the present system will be quite suggestive in a wider context, such as that of 2D colloidal liquids.

In fact, correspondence between the 2D dynamics of colloids and the non-ideal SFD may deserve serious consideration. Displacement correlations in 2D colloidal liquids [54] were recently calculated analytically with the method of the label variable [21]. A linear analysis of the transformed Dean–Kawasaki equation was found to suffice to explain displacement correlations at larger scales, while a phenomenological correction was needed for behavior at smaller scales. The 2D dynamics involve dilatational and rotational modes; the former modes change the local density and correspond to ψ(ξ,t) in SFD, while the latter (or, to be precise, their short-wave components) allow the particles to escape from the cage and correspond to $\delta \Xi_{i}\left(t\right)$ in this sense. More intuitively speaking, the overtaking event in Figure 3b can be understood as a small vortex involving two particles, namely 4 and 5.

The second issue concerns the approximation of treating $\overset{ˇ}{\psi }$ and $\delta \Xi_{i}$ in Equation (42) as uncorrelated, which seems to have made the behavior of Equations (43) and (44) quantitatively incorrect for shorter distances. While a quantitative test of Equation (44) is already given in Figure 6, the MSD in Equation (43) requires more discussion. The validity of Equation (43) could be checked by way of

(46) $$D_{\alpha }\overset{\mathrm{def}}{=}\lim_{t\to \infty }\frac{\langle R^{2}\rangle }{2t},$$

which can be computed numerically and compared with analytical predictions. It seems, however, that the $\nu_{\alpha }$-dependence of $D_{\alpha }$ is in dispute. Hahn and Kärger [23] asserted $D_{\alpha }\propto \nu_{\alpha }$, while Mon and Percus [24] claimed $D_{\alpha }\propto \nu_{\alpha }^{1/2}$. If Equation (43) is taken literally, it predicts that the longtime behavior of MSD is dominated by the second term on the right side and makes essentially the same prediction as Hahn and Kärger [23]; unfortunately, it contradicts the numerical results of Mon and Percus [24], at least for a certain range of $\nu_{\alpha }$. On the other hand, the reasoning about the origin of $D_{\alpha }\propto \nu_{\alpha }^{1/2}$ by Mon and Percus [24] is unsatisfactory, as it seems to lack connection with the collective motion.

As a possible scenario for reconciliation, we may conjecture that the correlation between $\overset{ˇ}{\psi }$ and $\delta \Xi_{i}$, ignored in the present theory, makes a difference to the short-range behavior of $\langle R_{i}R_{j}\rangle$ and in the MSD as its limiting case. It seems physically plausible that the collapse of smaller cages may be influenced by density fluctuations with long wavelengths which correlate $\overset{ˇ}{\psi }$ and $\delta \Xi_{i}$. If this correlation modifies the term containing *S* in Equation (43) so as to give

(47) R2ℓ02≃2πS+cναt(D*′t+2ναt

with some constant *c*, then it predicts $D_{\alpha }$ to be a sum of terms proportional to $\sqrt{\nu_{\alpha }}$ and $\nu_{\alpha }$. This form might be consistent at once with Hahn and Kärger [23] and with Mon and Percus [24], depending on the values of parameters.

All these issues originate from the phenomenological treatment of overtaking. Improvement upon the present analysis, going beyond the “Amida-kuji” or random exchange approximation, will need to be grounded on the integral in Equation (22) that gives $d\Xi_{i}/dt$. Its systematic treatment will allow calculation of $\nu_{\alpha }$, and it will also make it possible to take the correlation between $\overset{ˇ}{\psi }$ and $\Xi_{i}$ into account. The integrand in Equation (22) is given, in principle, by a solution of the Dean equation [32] for the single particle density, $\rho_{i}=\rho_{i}\left(x,t\right)$, and its flux, $Q_{i}$:

(48a) $$\begin{array}{c} \partial_{t}\rho_{i}\left(x,t\right)+\partial_{x}Q_{i}=0 \end{array}$$

(48b) $$\begin{array}{c} Q_{i}=-D\left[\partial_{x}\rho_{i}+\frac{\rho_{i}}{k_{B}T}\partial_{x}\sum_{j}V\left(x-X_{j}\left(t\right)\right)\right]+\rho_{i}f_{i}. \end{array}$$

Technically difficult though it might be, this strategy seems quite natural, as it is consistent with the treatment of ψ based on the Dean–Kawasaki equation (12). Development of this strategy for systematic treatment of overtaking effects on SFD will provide useful insights into cage-breaking events in 2D and 3D colloidal glasses.

## Abbreviations

The following abbreviations are used in this manuscript:

1D	one-dimensional
2D	two-dimensional
3D	three-dimensional
MSD	mean square displacement
SFD	single-file diffusion

## Appendix A. Two-Body Propagator in the Amida-Kuji Process

Here we outline solution of the master equation (39) for the two-body diffusion propagator in the Amida-kuji process. Note that most of the symbols in this Appendix (such as λ, μ and σ) are unrelated to the homonymous ones used in the main text.

To introduce some notation, we begin with a simpler problem of the one-body diffusion propagator governed by Equation (38). Its solution is found to be expressible in terms of

(A1) $$\Phi_{n}\left(s\right)\overset{\mathrm{def}}{=}\int_{-\pi }^{\pi }\exp\left[in\lambda +s(\cos\lambda -1)\right]\frac{d\lambda }{2\pi }=e^{-s}I_{n}\left(s\right)\;\left(n\in Z,\;s\in R\right),$$

where $I_{n}$ denotes the *n*-th modified Bessel function. This is obtained by looking for a solution in Fourier representation,

$$P\left(a,t\right)=\sum_{l}{\hat{P}}_{l}^{}\left(t\right)e^{ia\lambda },\;{\hat{P}}_{l}^{}\left(t\right)\propto e^{-\sigma t},\;\lambda =\frac{2\pi l}{N},$$

which is found to satisfy Equation (38) if $\sigma =2\nu_{\alpha }\left(1-\cos\lambda \right)$. The amplitude of ${\hat{P}}_{l}^{}$ is determined by the Fourier representation of the initial condition,

$$P\left(a,0\right)=\delta_{a,0}=\frac{1}{N}\sum_{l}e^{ia\lambda },$$

so that the solution is

(A2) $$P\left(a,t\right)=\frac{1}{N}\sum_{l}e^{ia\lambda -\sigma t}\to \Phi_{a}\left(2\nu_{\alpha }t\right)$$

in the limit of N→∞. This solution allows calculating

$$\langle {\left[\delta \Xi_{i}\left(t\right)\right]}^{p}\rangle =\sum_{a=-\infty }^{+\infty }a^{p}P\left(a,t\right);$$

in particular, for p=2 we obtain Equation (37).

The two-body problem in Equation (39) is more complicated. As a counterpart of Δ=j−i, we introduce d=b−a. Although a general solution could be sought in Fourier representation as $P_{a,b}=\sum_{l}{\tilde{P}}_{l,d}\left(t\right)e^{ia\lambda }$, here we concentrate on the mode with l=0, introducing

(A3) $$Q_{d}=Q\left(\Delta ,d,t\right)\overset{\mathrm{def}}{=}\sum_{a}P\left(i,i+\Delta ,0;a,a+d,t\right);$$

this is sufficient for the present purpose, as the knowledge of $Q_{d}$ will allow us to calculate

(A4) $$\langle {\left[\delta \Xi_{j}\left(t\right)-\delta \Xi_{i}\left(t\right)\right]}^{p}\rangle =\sum_{d}{\left(d-\Delta \right)}^{p}Q\left(\Delta ,d,t\right).$$

In terms of $Q_{d}$, the master equation (39) is rewritten as

(A5) $$\partial_{t}Q_{d}=2\nu_{\alpha }\left(Q_{d+1}+Q_{d-1}-2Q_{d}\right)+\left(\delta_{d,1}+\delta_{d,-1}\right)\nu_{\alpha }\left(Q_{1}+Q_{-1}\right)\;\left(d\neq 0\right),$$

while $Q_{0}$ vanishes identically. The initial condition can be expressed as

(A6) $${\left.Q_{d}\right|}_{t=0}=Q\left(\Delta ,d,0\right)=\delta_{\Delta ,d}=\frac{1}{N}\sum_{m}e^{i(d-\Delta )\mu }$$

where μ=2πm/N, the summation ranges from −N/2 to N/2 (with the endpoints included by half), and Δ is taken to be positive without loss of generality.

To solve Equation (A5), we assume

(A7) $$Q_{d}=\left\{\begin{array}{cc} \frac{1}{N}\sum_{m}A_{m}e^{id\mu }e^{-\sigma_{m}t} & \left(d>0\right)\\ 0 & \left(d=0\right)\\ \frac{1}{N}\sum_{m}B_{m}e^{id\mu }e^{-\sigma_{m}t} & \left(d<0\right) \end{array}\right.$$

with $A_{-m}=A_{m}^{*}$ and $B_{-m}=B_{m}^{*}$. By considering the cases of $\left|d\right|\geq 2$ in Equation (A5), we find

(A8) $$\sigma_{m}=\sigma_{-m}=2\nu_{\alpha }\left(2-\varepsilon -\varepsilon^{*}\right)$$

with $\varepsilon \overset{\mathrm{def}}{=}e^{i\mu }$. Subsequently, evaluation of Equation (A5) for d=±1 gives

(A9a) $$\begin{array}{cc} \left(2-\varepsilon \right)A_{m}+\left(2-\varepsilon^{*}\right)A_{-m} & =\varepsilon^{*}B_{m}+\varepsilon B_{-m} \end{array}$$

(A9b) $$\begin{array}{cc} \left(2-\varepsilon^{*}\right)B_{m}+\left(2-\varepsilon \right)B_{-m} & =\varepsilon A_{m}+\varepsilon^{*}A_{-m}, \end{array}$$

which also implies $A_{m}+A_{-m}=B_{m}+B_{-m}$.

Although the initial condition (A6) seems to suggest $A_{m}=B_{m}=\varepsilon^{-\Delta }$, this naive choice does not satisfy Equations (A9). Instead, we assume

(A10) $$A_{m}=\varepsilon^{-\Delta }+C,\;B_{m}=\varepsilon^{-\Delta }+C^{*},$$

where C=C(ε) is a polynomial of ε (consisting of terms with non-negative powers only) so that

$$\sum_{m}C\left(\varepsilon \right)\varepsilon^{d}=0$$

for d>0. Substituting Equation (A10) into Equations (A9) yields

(A11) $$2\left(1-\varepsilon \right)C+2\left(1-\varepsilon^{*}\right)C^{*}=\left(-2+\varepsilon +\varepsilon^{*}\right)\left(\varepsilon^{\Delta }+\varepsilon^{-\Delta }\right),$$

which is satisfied if we choose

(A12) $$C=\frac{1}{2}\left(\varepsilon^{-1}-1\right)\varepsilon^{\Delta }.$$

Note that this is indeed a polynomial, since Δ≥1. Thus $Q_{d}$ is obtained in the form of Equation (A7), with $A_{m}$ and $B_{m}$ given by Equations (A10) and (A12), where $\varepsilon =e^{i\mu }=e^{2\pi im/N}$.

In the limit of N→∞, the summations in Equation (A7) are evaluated as integrals, expressible in terms of $\Phi_{n}\left(s\right)$ in Equation (A1). The result reads

(A13) $$Q_{d}=\left\{\begin{array}{cc} \Phi_{d-\Delta }\left(4\nu_{\alpha }t\right)-\frac{1}{2}\Phi_{d+\Delta }\left(4\nu_{\alpha }t\right)+\frac{1}{2}\Phi_{d+\Delta -1}\left(4\nu_{\alpha }t\right) & \left(d>0\right)\\ 0 & \left(d=0\right)\\ \frac{1}{2}\Phi_{d-\Delta }\left(4\nu_{\alpha }t\right)+\frac{1}{2}\Phi_{d-\Delta +1}\left(4\nu_{\alpha }t\right) & (d<0). \end{array}\right.$$

This result allows us to calculate the moments of $\delta \Xi_{j}-\delta \Xi_{i}$ in Equation (A4). In particular, using the second moment, we can also calculate $\langle \delta \Xi_{i}\delta \Xi_{j}\rangle$ as

$$\langle \delta \Xi_{i}\delta \Xi_{j}\rangle =\frac{1}{2}\left[\langle \delta \Xi_{i}^{2}\rangle +\langle \delta \Xi_{j}^{2}\rangle -\langle {\left(\delta \Xi_{j}-\delta \Xi_{i}\right)}^{2}\rangle \right],$$

where $\langle \delta \Xi_{i}^{2}\rangle =\langle \delta \Xi_{j}^{2}\rangle =2\nu_{\alpha }t$ according to Equation (37). After some rearrangement with the recurrence formula for the modified Bessel function, we arrive at Equation (45).

## Appendix B. Numerical Evaluation of the Hopping Rate

Within the approximation of the present work, the hopping rate $\nu_{\alpha }$ is regarded as a constant whose value depends on the control parameters of the system. The problem of how to specify $\nu_{\alpha }$ might be trivial if the lattice dynamics were adopted instead of the present system with the continuous space, because the probability of overtaking, i.e., the ratio of $\nu_{\alpha }$ to the collision frequency, would be given as a part of dynamical rules governing the lattice system. More consideration is required in the present case, because overtaking is not given as an elementary process but determined as a result of the Langevin dynamics regulated by the interaction potential V(r) with r∈R.

For the present, we have determined the function $\nu_{\alpha }=\nu_{\alpha }\left(\rho_{0},V_{\max}\right)$ by solving Equation (2) numerically, with V(r) given in Equation (5); for numerical ease, the mass is chosen to be finite so that $m/\mu :\sigma^{2}/D=1:1$. For each run with some specific values of $\left(\rho_{0},V_{\max}\right)$, we counted overtaking events that had occurred since the time 0, and plotted the cumulative number of overtaking, divided by *N*, against *t*. Thus we obtain a plot analogous to Figure 4(a) in Ref. [25] that can be fitted with a straight line, whose slope gives $\nu_{\alpha }$. The values of $\nu_{\alpha }$, collected in this way as a function of $\left(\rho_{0},V_{\max}\right)$, are fitted with an Arrhenius-like expression, with a prefactor that depends both on $V_{\max}$ and $\rho_{0}$ [44]:

(A14) $$\nu_{\alpha }=D\left(a_{0}\frac{\rho_{0}}{\sigma }+a_{1}\rho_{0}^{2}\;\beta V_{\max}\right)e^{-\beta V_{\max}},\;\beta =\frac{1}{k_{B}T},$$

where $a_{0}\approx 1/2$ and $a_{1}\approx 1/6$.

The numerical values of $\nu_{\alpha }$ reported here, however, should not be regarded as decisive. We must be cautious about difficulty in precise numerical calculation of rare events such as overtaking. In comparison to other quantities, computation of $\nu_{\alpha }$ may be sensitive to the choice of the numerical scheme, and in the present calculation, based on a Verlet-like scheme, the computed values of $\nu_{\alpha }$ seem to depend on the ratio m/μ:$\sigma^{2}/D$. A systematic study of this issue will be expected in the future.
